# Phase-resolved Higgs response in superconducting cuprates

**DOI:** 10.1038/s41467-020-15613-1

**Published:** 2020-04-14

**Authors:** Hao Chu, Min-Jae Kim, Kota Katsumi, Sergey Kovalev, Robert David Dawson, Lukas Schwarz, Naotaka Yoshikawa, Gideok Kim, Daniel Putzky, Zhi Zhong Li, Hélène Raffy, Semyon Germanskiy, Jan-Christoph Deinert, Nilesh Awari, Igor Ilyakov, Bertram Green, Min Chen, Mohammed Bawatna, Georg Cristiani, Gennady Logvenov, Yann Gallais, Alexander V. Boris, Bernhard Keimer, Andreas P. Schnyder, Dirk Manske, Michael Gensch, Zhe Wang, Ryo Shimano, Stefan Kaiser

**Affiliations:** 1 Max Planck Institute for Solid State Research, Heisenbergstr. 1, 70569 Stuttgart, Germany; 2 4th Physics Institute, University of Stuttgart, 70569 Stuttgart, Germany; 3 Department of Physics, University of Tokyo, Hongo, Tokyo 113-0033 Japan; 4 Helmholtz-Zentrum Dresden-Rossendorf, Bautzner Landstr. 400, 01328 Dresden, Germany; 5 Laboratoire de Physique des Solides (CNRS UMR 8502), Bâtiment 510, Université Paris-Saclay, 91405 Orsay, France; 6 University of Groningen, 9747 AG Groningen, Netherlands; 7 Technische Universität Berlin, Institut für Optik und Atomare Physik, Strasse des 17. Juni 135, 10623 Berlin, Germany; 8 Laboratoire Matériaux et Phénomènes Quantiques (UMR 7162 CNRS), Université de Paris, Bâtiment Condorcet, 75205 Paris Cedex 13, France; 9 German Aerospace Center (DLR), Institute of Optical Sensor Systems, Rutherfordstrasse 2, 12489 Berlin, Germany; 10 Cryogenic Research Center, University of Tokyo, Hongo, Tokyo, 113-0032 Japan; 11 Present Address: Stewart Blusson Quantum Matter Institute, University of British Columbia, Vancouver, BC V6T 1Z4 Canada; 12 Present Address: Institute of Physics II, University of Cologne, 50937 Cologne, Germany

**Keywords:** Superconducting properties and materials, High-harmonic generation

## Abstract

In high-energy physics, the Higgs field couples to gauge bosons and fermions and gives mass to their elementary excitations. Experimentally, such couplings can be inferred from the decay product of the Higgs boson, i.e., the scalar (amplitude) excitation of the Higgs field. In superconductors, Cooper pairs bear a close analogy to the Higgs field. Interaction between the Cooper pairs and other degrees of freedom provides dissipation channels for the amplitude mode, which may reveal important information about the microscopic pairing mechanism. To this end, we investigate the Higgs (amplitude) mode of several cuprate thin films using phase-resolved terahertz third harmonic generation (THG). In addition to the heavily damped Higgs mode itself, we observe a universal jump in the phase of the driven Higgs oscillation as well as a non-vanishing THG above *T*_c_. These findings indicate coupling of the Higgs mode to other collective modes and potentially a nonzero pairing amplitude above *T*_c_.

## Abstract (web summary)

Interaction between Cooper pairs and other collective excitations may reveal important information about the pairing mechanism. Here, the authors observe a universal jump in the phase of the driven Higgs oscillations in cuprate thin films, indicating the presence of a coupled collective mode, as well as a nonvanishing Higgs-like response at high temperatures, suggesting a potential nonzero pairing amplitude above *T*c.

## Introduction

For field theories with U(1) continuous symmetry and respecting Lorentz invariance or particle-hole symmetry, spontaneous symmetry breaking gives rise to an order parameter with two orthogonal collective modes: the Goldstone mode along the azimuthal direction, and the Higgs mode along the radial direction^1^. In superconductors, discussions about the Higgs mode and the Higgs mechanism precede those in high-energy physics^2,3^. However, their significance to superconductivity has been more slowly appreciated. Experimental detection of the Higgs mode has also been hampered by its lack of electric and magnetic dipole moments in most superconductors. Recently, it was proposed that the Higgs mode may reveal the superconducting gap symmetry and multiplicity^4–6^, unveil coupled collective modes^7^, or explain aspects of light-induced superconductivity^8^. Novel methods for exciting and detecting the superconducting Higgs mode were also demonstrated using ultrafast terahertz techniques in the meantime^9,10^. Specifically, free oscillations of the Higgs mode, with its characteristic frequency of 2Δ, can be launched by an ultrashort terahertz pulse quenching the free energy of the order parameter^9^. Alternatively, it could also be periodically driven at 2*ω* through nonlinear coupling between the electromagnetic vector potential **A**(*ω*) and the superconducting condensate^10–12^. The resulting free (driven) Higgs oscillation manifests itself in terahertz transmissivity as an oscillation at 2Δ (2*ω*). In the latter scenario, the 2ω oscillation of the condensate interacts with the driving field **A**(*ω*), leading to sum frequency generation or third harmonic generation (THG), which is resonantly enhanced when 2*ω* = 2Δ(*T*)^10,13^.

While both free and driven Higgs oscillations have been demonstrated in *s*-wave superconductors, the Higgs mode of *d*-wave superconductors is more complex. The continuous variation of Δ between 0 and Δ^max^ along different directions of the Brillouin zone leads to strong dephasing of the mode. This is compounded by the existence of quasiparticle excitations at arbitrarily low energies, which provide rapid decay channels and significantly damp the mode^14^. Terahertz pump optical probe experiments on Bi_2_Sr_2_CaCu_2_O_8+x_ single crystals have provided the first experimental evidence for an isotropic Higgs response of *d*-wave superconductors in the form of an |**A**|^2^ response in the condensate’s optical reflectivity to a monocycle terahertz pulse^15^. On the other hand, periodically driving the Higgs oscillation would provide useful phase information that may indicate resonance and coupling to other modes. Such an experiment requires a multicycle, carrier-envelope phase-stable terahertz source with a narrow bandwidth and high electric field strength, which is provided by the TELBE superradiant undulator source at HZDR^16^. Using this facility, we investigate the THG response of optimally-doped La_1.84_Sr_0.16_CuO_4_ (*T*_c_ = 45 K), DyBa_2_Cu_3_O_7−x_ (*T*_c_ = 90 K), YBa_2_Cu_3_O_7−x_ (*T*_c_ = 88 K), and overdoped Bi_2_Sr_2_CaCu_2_O_8+x_ (*T*_c_ = 65 K) thin films (Supplementary Note 1). Our experiment is performed with 0.7 THz driving frequency, with an electric field up to ~50 kV cm^−1^ (Supplementary Note 2). In all of these samples, we observe an increase in THG intensity (*I*_TH_) below *T*_c_ that is consistent with a heavily damped Higgs oscillation. In addition, in LSCO(OP45), DyBCO(OP90) and YBCO(OP88), a universal jump in the relative phase between the THG response and the linear drive (*Φ*_TH_) at *T* < *T*_c_ is observed, signifying the coupling of the Higgs mode to another collective mode. We also observe a nonzero *I*_TH_ persisting above *T*_c_ in all of the samples, which may indicate preformed Cooper pairs above *T*_c_.

## Results

### THG response of d-wave superconductors

**Fig. 1:**
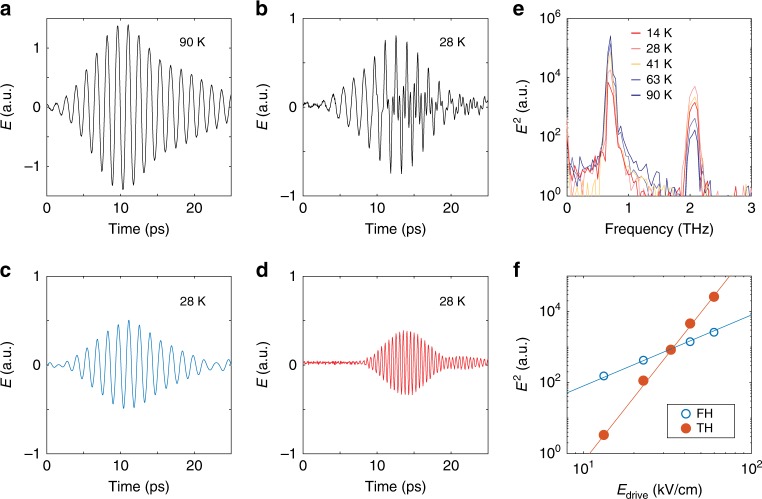
THG from driven Higgs oscillation in LSCO(OP45). **a**, **b** Terahertz field transmitted through LSCO(OP45) at 90 K and 28 K. A 2.1 THz bandpass filter is placed after the sample to suppress the 0.7 THz transmission (Supplementary Note 2). **c**, **d** 0.7 THz fundamental harmonic (FH) and 2.1 THz third harmonic (TH) extracted from **b** using 1.4 THz FFT low pass and high pass filters. **e** FFT power spectrum of the transmitted field at selected temperatures across *T*_c_ = 45 K. **f** Transmitted FH and TH power versus incoming FH field at 28 K. Solid lines are guides-to-the-eye with a slope of 2 and 6.

**Fig. 2:**
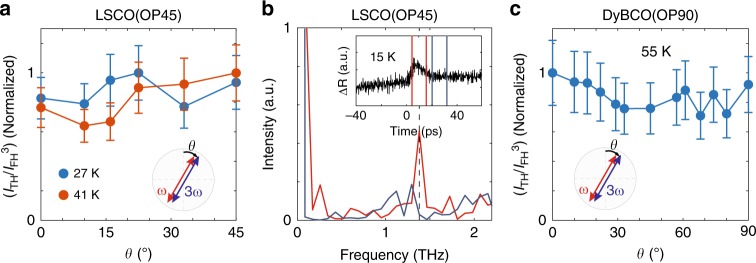
Signature of the driven Higgs oscillations in transient response and polarization dependence. **a** THG intensity normalized by the parallel transmitted FH power (*I*_TH_/*I*_FH_^3^) for LSCO(OP45) as a function of *θ*, the angle from the Cu–O bond direction. THG from the Higgs oscillation is expected to be isotropic, while THG from charge density fluctuation (CDF) is expected to be anisotropic. **b** Transient reflectivity of LSCO(OP45) is measured with an 80 fs optical pulse while it is pumped with the 0.7 THz multicycle terahertz pulse. The change in reflectivity, ΔR, as a function of delay between the pump pulse and the probe pulse is shown in the inset. Main figure shows the FFT power spectrum of the relevant time intervals marked in the inset. ΔR exhibits a 1.4 THz (black dotted line) modulation while the pump pulse is on (red line) due to the |**A** | ^2^ coupling of the condensate to the terahertz drive. The 1.4 THz peak becomes indistinguishable after the pump pulse is gone (blue line). **c** THG polarization dependence for DyBCO(OP90). The error bars represent standard deviation due to pulse-to-pulse intensity fluctuation.

To illustrate the THG response of *d*-wave superconductors, first we show terahertz transmission through LSCO(OP45). As Fig. 1a–d shows, while the residual fundamental harmonic (FH) dominates the terahertz transmission above *T*_c_, a large amplitude of third harmonic (TH) becomes visible below *T*_c_. Moreover, FH transmission (*I*_FH_) monotonically decreases with decreasing temperature as shown in Fig. 1e. In comparison, TH intensity (*I*_TH_) exhibits a maximum below *T*_c_. In the context of superconductors, the nonlinear Meissner effect^17,18^, charge density fluctuations (CDF)^19^, and Higgs oscillations have been previously reported or discussed to give rise to THG. Unlike what is observed in this study, the nonlinear Meissner effect manifests as a narrow peak around *T*_*c*_ in the 3^rd^ order nonlinear current. It is often discussed in terms of nonlinear Josephson current and might be probing the phase response of weakly connected superconducting islands^1^. On the other hand, studies on *s*-wave and *d*-wave superconductors have suggested an anisotropic response from CDF, while THG from the fully symmetric (A_1g_) Higgs oscillation is expected to be isotropic^15,19–21^. To distinguish between CDF and the A_1g_ Higgs response, we performed THG polarization dependence measurements. An isotropic response is found to dominate (Fig. 2a, c). In addition, we performed terahertz pump optical probe (TPOP) measurements similar to Reference 15. An |**A**(*ω*)|^2^ response to the **A**(*ω*) driving field is also seen in the condensate’s optical reflectivity (Fig. 2b). These results may not uniquely identify, but are consistent with a driven Higgs response to the multicycle terahertz pulse and its role in THG below *T*_c_. The THG polarization dependence indicates that there might be a finite contribution from other sources such as CDF or additional nonlinear mechanisms not yet discussed or experimentally evidenced. Therefore, a full understanding of the different sources of THG in addition to the driven Higgs oscillations requires further experimental and theoretical efforts. For instance, it will be interesting to investigate the systematic doping dependence of the anisotropic response in THG and TPOP experiments to quantify the relative contributions between different symmetry components^15^. Finally, to ensure that the Higgs oscillation stays in the perturbative excitation regime, we performed fluence dependence measurements. An excellent agreement with the expected *I*_TH_ ∝ *I*_FH_^3^ dependence is observed (Fig. 1f) (Supplementary Note 6).

### Temperature dependence of THG

**Fig. 3:**
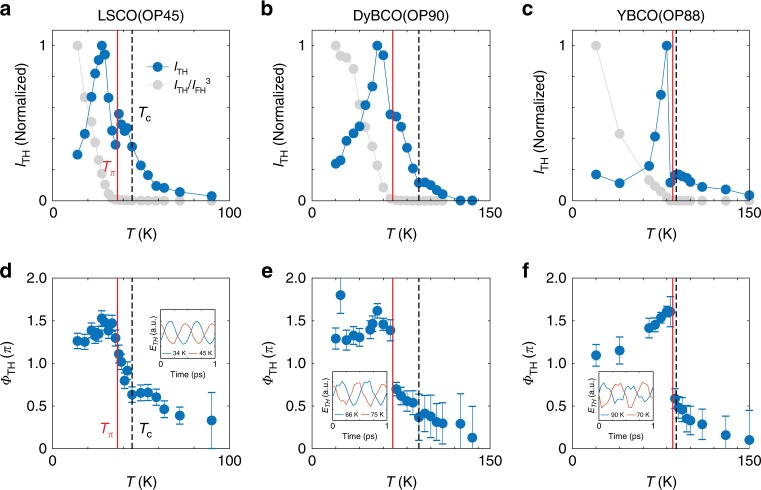
Temperature dependence of TH intensity (*I*_TH_) and relative phase (*Φ*_TH_) from optimally-doped cuprates. **a**–**c** Temperature dependence of TH intensity, *I*_TH_ (blue), and normalized TH intensity, *I*_TH_/*I*_FH_^3^ (gray), in LSCO(OP45), DyBCO(OP90) and YBCO(OP88). **d**–**f** Temperature dependence of the relative phase between the TH response and the FH drive, extracted from waveforms such as those in Fig. 1c, d. Inset shows representative TH waveforms across the π phase jump temperature (*T*_π_). The dotted line (black) denotes *T*_c_ and the solid line (red) denotes *T*_π_. The error bars represent the two sigma uncertainty from fitting the phase of TH and FH waveforms (Supplementary Note 4).

To look for potential resonance of the Higgs oscillation at 2*ω* = 2Δ(*T*), we performed detailed temperature dependence measurements as shown in Fig. 3a–c. In LSCO(OP45) we observe a peak in *I*_TH_ near 0.6*T*_c_, as well as a smaller peak around 0.9*T*_c_. DyBCO(OP90) exhibits a similar peak in *I*_TH_ near 0.6*T*_c_. In comparison, YBCO(OP88) exhibits a sharp peak in *I*_TH_ near 0.9*T*_c_ and a hump around *T*_c_. In BSCCO(OD65), a continuously increasing *I*_TH_ with decreasing temperature is observed (Supplementary Note 7). A careful examination of the transmitted *I*_FH_ reveals that the main peak in *I*_TH_ originates from the competition between a growing nonlinear response of the Higgs oscillation and an increasing screening of the driving field as temperature decreases (Supplementary Note 3). Therefore, the main peak is not a resonance feature. In fact, for optimally-doped cuprates, we expect Δ(*T* = 0) ≳ 20 meV and a steep onset of Δ at *T*_c_. Therefore, the 2*ω* = 2Δ(*T*) resonance, if present, is expected to be satisfied immediately below *T*_c_ for *ω* = 0.7 THz (~3 meV). Moreover, as the Higgs mode of *d*-wave systems is heavily damped, a resonance peak in *I*_TH_ is expected to be significantly broadened. This motivates us to investigate the phase of the driven Higgs oscillation, which is expected to exhibit a prominent change across resonance even in the presence of strong damping (Supplementary Note 8).

### Phase evolution of THG below *T*_c_

In Fig. 3d–f, we extracted the relative phase (*Φ*_TH_) of the TH with respect to the FH signal (Supplementary Note 4). Despite dissimilar features in *I*_TH,_ all three samples exhibit a similar response in *Φ*_TH._ In particular, an abrupt jump of nearly π happens at a temperature *T*_π_ < *T*_c_. In YBCO(OP88), *T*_π_ is in the range where 2*ω* = 2Δ(*T*) is expected to be satisfied. However, such a sharp phase jump is again inconsistent with the resonance of a heavily damped collective mode. In LSCO(OP45) and DyBCO(OP90), *T*_π_ is significantly lower than *T*_c_. In light of these, we do not attribute the universal phase jump to the 2*ω* = 2Δ(*T*) Higgs resonance. A more striking evidence for such an interpretation comes from the direction of the phase jump. Since the low-temperature regime corresponds to driving below resonance (2*ω* < 2Δ(*T*)) and the high-temperature regime to driving above resonance (2*ω* > 2Δ(*T*)), a resonance-like phase jump should evolve positively with temperature whereas the observed *Φ*_TH_ jumps negatively with temperature.

## Discussion

**Fig. 4:**
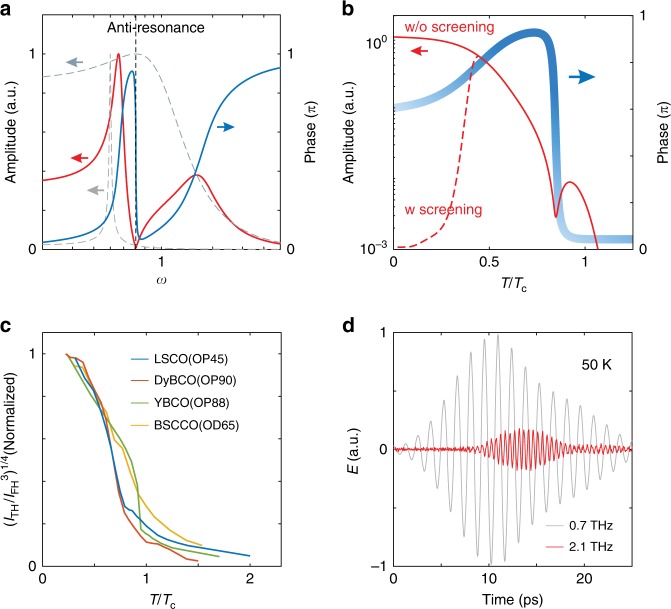
Driven coupled oscillators model and finite THG above *T*_c_. **a** The dotted lines depict the amplitude response of a critically damped harmonic oscillator and an underdamped harmonic oscillator as the driving frequency *ω* is varied. When these two oscillators are coupled, the coupled system retains two resonances but also develops an anti-resonance. Across the anti-resonance (vertical dotted line), the amplitude (solid red line) of the driven oscillator goes through a minimum while its phase (solid blue line) jumps negatively with the driving frequency. **b** Assuming the driving frequency is fixed as in our experiment, but that the resonance frequency of the two oscillators varies with temperature as Δ(*T*) and δΔ(*T*) (δ < 1), the response of the coupled oscillators system is shown as a function of *T*. The anti-resonance in **a** is recaptured: the amplitude of the Higgs oscillation (thin red line) goes through a minimum while its phase (thick blue line) jumps negatively with *T*. The dotted line illustrates the effect of screening, which is to reduce the driving force and hence the amplitude at lower *T*. **c** Temperature dependence of (*I*_TH_/*I*_FH_^*3*^)^1/4^, which remains finite above *T*_c_. (*I*_TH_/*I*_FH_^*3*^)^1/4^ is theoretically predicted to be ∝ Δ away from resonance. **d** FH and TH components extracted from the transmitted waveform from LSCO(OP45) at 50 K > *T*_c_.

To obtain an intuitive understanding of the phase response, we look at a driven coupled harmonic oscillators model. Whereas isolated oscillators exhibit a maximum in their amplitude and a positive phase jump ≲ π across resonance^13^ (Supplementary Note 8), the coupled oscillators system develops an anti-resonance in addition to resonances. This manifests as a minimum in the amplitude of the driven oscillator, simultaneous with a phase jump in the negative direction (Fig. 4a). To more closely model our experiment, we fix the driving frequency but allow the energetics of the oscillators to depend on temperature (Fig. 4b) (Supplementary Note 8). By choosing their resonance frequencies as Δ(*T*) and δΔ(*T*) (δ < 1), where Δ(*T*) = $\sqrt {n_{\mathrm{s}}(T)}$ (*n*_s_ is the experimentally measured superfluid density in LSCO(OP45)), the model recaptures the essential features of LSCO(OP45) (Fig. 3a,d). In YBCO(OP88) a smaller dip in *I*_TH_(*T*) is seen while in DyBCO(OP90) a kink in *I*_TH_(*T*) is observed at the *T*_π_. This could be due to the sharpness of the phase jump in these two samples, causing the dip in *I*_TH_(*T*) to be very narrow in temperature and buried between the measured temperature points. In the framework of the coupled oscillators model, this suggests that the coupled mode is less damped in the bilayer systems compared to the single-layer LSCO(OP45). A classical Fresnel analysis of the thin film effects excludes the possibility of the phase jump coming from linear shifts in *Φ*_FH_(*T*) and *Φ*_TH_(*T*) (Supplementary Note 5).

While our classical toy model does not aim to explain the microscopic origin of the observed THG response, it allows us to learn about the dynamics and energetics of the coupled mode. It suggests that the coupled mode is underdamped and has an energy scale comparable to the Higgs mode. Moreover, the energy of this mode and/or its coupling to the Higgs mode depends on temperature. Within these constraints, potential candidates for the coupled collective mode include paramagnons, collective charge fluctuations of the incipient charge density wave (CDW) order, or phonons. In particular, coupling between the Higgs mode and CDW excitations has been observed in NbSe_2_ and 2*H*-TaS_2_ superconductors^22,23^. In cuprates, charge order exhibits a similar energy scale as the superconducting gap^24^. Evidence for the incipient charge order in a wide range of the phase diagram has also been reported^25^. On the other hand, paramagnons are strongly renormalized in the superconducting state into a sharp underdamped resonance mode in a similar energy window as the superconducting gap^26^. They are prominent candidates for mediating Cooper pairing in high-*T*_c_ superconductors. Last but not least, strong electron-phonon coupling may also contribute a phononic character to the superconducting order parameter^27^, leading to new amplitude mode(s) in the Higgs response^7^. Other exotic but potential candidates include the Bardasis-Schrieffer mode^28,29^ and the anisotropic A_2g_/B_1g_ Higgs mode^4,6^ (Supplementary Note 9), which are also collective modes of the superconducting order parameter. Future doping and magnetic field dependence studies may shed light on the identity of the coupled mode.

While the identity of the coupled mode demands further scrutiny, our experiment also reveals a non-vanishing THG response above *T*_c_ in all samples. In Fig. 4c, we plot the temperature dependence of (*I*_TH_/*I*_FH_^3^)^1/4^, which is theoretically predicted to be ∝ Δ away from resonance^13^. Indeed, (*I*_TH_/*I*_FH_^3^)^1/4^ exhibits an order parameter-like temperature dependence below *T*_c_. Surprisingly, it remains nonzero up to *T* > 1.5*T*_c_, similar to the temperature regime where superconducting fluctuations are observed in Nernst effect measurements^30^. This is more clearly illustrated by the transmitted TH waveform from LSCO(OP45) at *T* = 50 K > *T*_c_ (Fig. 4d). Our observation may indicate preformed Cooper pairs in cuprates without global phase coherence, or the intense terahertz field might enforce phase coherence above *T*_c_^12^. The pseudogap, and its various ordered phases including the CDW order, may also play a role in THG above *T*_c_^31,32^.

While the coupled oscillators model offers an intuitive explanation for the unexpected phase jump in THG in several families of cuprates, it is a classical toy model and calls for a full quantum mechanical treatment of the subject to provide deeper insight. In parallel, future magnetic field and doping dependence investigations may further help unveil the nature of the coupled mode and the non-vanishing THG response above *T*_c_. Sweeping the driving frequency may also help distinguish the different scenarios of *d*-wave gap closing or filling as temperature approaches *T*_c_. While these initiatives are underway, our technique may also serve as a probe for non-equilibrium superconductivity^33,34^. With so many interesting prospects, we see a bright future for phase-resolved spectroscopy of collective modes in superconductors and beyond^35–38^.

## Methods

### Sample growth and characterization

The LSCO(OP45) and DyBCO(OP90) samples were grown by molecular beam epitaxy (MBE), and the YBCO(OP88) sample was grown by pulsed laser deposition (PLD) at the Max Planck Institute for Solid State Research. The LSCO(OP45) sample is 80 nm-thick on a LaSrAlO_4_ (LSAO) substrate. The DyBCO(OP90) sample is 70 nm-thick on a (LaAlO_3_)_0.3_(Sr_2_TaAlO_6_)_0.7_ (LSAT) substrate. The YBCO(OP88) sample is 200 nm-thick on a NdGaO_3_ (NGO) substrate. The BSCCO(OD65) sample was grown by sputtering technique at Laboratoire de Physique des Solides. The BSCCO(OD65) sample is 160 nm thick on a MgO substrate. As shown in Supplementary Note 1, *T*_c_ is determined from mutual inductance measurement for LSCO(OP45), DyBCO(OP90) and YBCO(OP88). *T*_c_ of BSCCO(OD65) is determined from the drop in magnetic moment from SQUID measurement under zero-field cooling. We define *T*_c_ as the onset of the drop in mutual inductance and magnetic moment during cooling.

### THG experiment

The majority of the data presented in this study are measured using the experimental setup shown in Supplementary Note 2. For fluence dependence measurements, we add an additional 1.93 THz bandpass filter (BPF) before Polarizer 3 (P3) to suppress the fundamental harmonic (FH). For temperature dependence of third harmonic (TH) in BSCCO(OD65), we also add an additional 1.9 THz BPF before P3. For electro-optical sampling we used a 2 mm ZnTe crystal and 100 fs gate pulse with 800 nm central wavelength. Accelerator-based THz pump and the laser gating pulse have a timing jitter characterized by a standard deviation of ~20 fs. Synchronization was achieved through pulse-resolved detection.

## Supplementary information

Supplementary Information

## Data Availability

The data that support the findings of this study are available from the first author and the corresponding authors upon reasonable request. The raw pre-sorted and pre-binned data that allow statistical analysis is available at 10.14278/rodare.277 together with the software tools that were used for data treatment. Further requests on data treatment should be sent to HZDR via S.Ko.

## Code availability

The numerical code used to calculate the results for this work is available from H.C. and L.S. upon reasonable request.
