# Supplementary material for: Phase-resolved Higgs response in superconducting cuprates
Source: Nat Commun. 2020 Apr 14;11:1793. doi: 10.1038/s41467-020-15613-1 (PMC7156672; doi:10.1038/s41467-020-15613-1)
Supplement: Supplementary file 1 — Supplementary Information [file 41467_2020_15613_MOESM1_ESM.pdf]

Supplementary Information

Phase-resolved Higgs response in superconducting cuprates

Hao Chu[1,2], Min-Jae Kim[1,2], Kota Katsumi[3], Sergey Kovalev[4], Robert David Dawson[1], Lukas Schwarz[1], Naotaka Yoshikawa[3], Gideok Kim[1], Daniel Putzky[1], Zhi Zhong Li[5], Hélène Raffy[5], Semyon Germanskiy[4], Jan-Christoph Deinert[4], Nilesh Awari[4,6], Igor Ilyakov[4], Bertram Green[4], Min Chen[4,7], Mohammed Bawatna[4], Georg Christiani[1], Gennady Logvenov[1], Yann Gallais[8], Alexander V. Boris[1], Bernhard Keimer[1], Andreas Schnyder[1], Dirk Manske[1], Michael Gensch[7,9], Zhe Wang[4], Ryo Shimano[3,10], Stefan Kaiser[1,2]

[1]*Max Planck Institute for Solid State Research, Heisenbergstr. 1, 70569 Stuttgart, Germany*

[2]*4th Physics Institute, University of Stuttgart, 70569 Stuttgart, Germany*

[3]*Department of Physics, University of Tokyo, Hongo, Tokyo,113-0033, Japan*

[4]*Helmholtz-Zentrum Dresden-Rossendorf, Bautzner Landstr. 400, 01328 Dresden, Germany*

[5]*Laboratoire de Physique des Solides (CNRS UMR 8502), Bâtiment 510, Université Paris-Saclay, 91405 Orsay, France*

[6]*University of Groningen, 9747 AG Groningen, Netherlands*

[7]*Technische Universität Berlin, Institut für Optik und Atomare Physik, Strasse des 17. Juni 135, 10623 Berlin, Germany*

[8]*Laboratoire Matériaux et Phénomènes Quantiques (UMR 7162 CNRS), Université de Paris, Bâtiment Condorcet, 75205 Paris Cedex 13, France*

[9]*German Aerospace Center (DLR), Institute of Optical Sensor Systems, Rutherfordstrasse 2, 12489 Berlin, Germany*

[10]*Cryogenic Research Center, University of Tokyo, Hongo, Tokyo,113-0032, Japan*

Supplementary Note 1. Sample growth and characterization

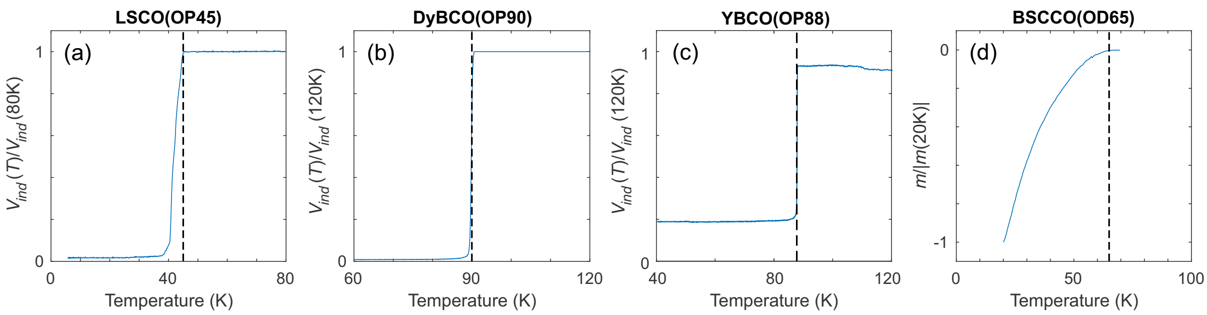

Supplementary Figure 1 Experimental determination of T_c in (a) LSCO (b) DyBCO and (c) YBCO by mutual inductance measurements and in (d) BSCCO by SQUID measurement. The mutual inductance results are normalized to their value above T_c. In BSCCO, the magnetic moment of the sample starts to drop at T_c. The drop is normalized to the magnetic moment at 20 K. Dotted line indicates T_c.

The LSCO(OP45) and DyBCO(OP90) samples were grown by molecular beam epitaxy (MBE), and the YBCO(OP88) sample was grown by pulsed laser deposition (PLD) at the Max Planck Institute for Solid State Research. The LSCO(OP45) sample is 80 nm-thick on a $LaSrAlO_4$ (LSAO) substrate. The DyBCO(OP90) sample is 70 nm-thick on a $(LaAlO_3)_{0.3}(Sr_2TaAlO_6)_{0.7}$ (LSAT) substrate. The YBCO(OP88) sample is 200 nm-thick on a $NdGaO_3$ (NGO) substrate. The BSCCO(OD65) sample was grown by sputtering technique at Laboratoire de Physique des Solides. The BSCCO(OD65) sample is 160 nm thick on a MgO substrate.

As shown in Supplementary Figure 1, T_c is determined from mutual inductance measurement for LSCO(OP45), DyBCO(OP90) and YBCO(OP88). T_c of BSCCO(OD65) is determined from the drop in magnetic moment from SQUID measurement under zero-field cooling. We define T_c as the onset of the drop in mutual inductance and magnetic moment during cooling.

Supplementary Note 2. Experimental setup

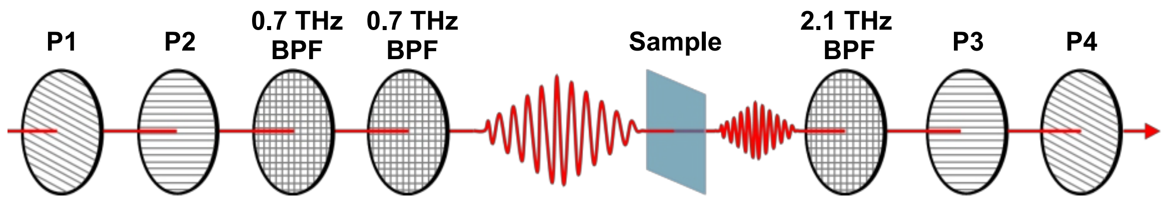

Supplementary Figure 2.1 Experimental setup for the Higgs third harmonic generation experiment. P1-P4: wire-grid polarizers. BPF: bandpass filter.

The majority of the data presented in this study are measured using the experimental setup shown in Supplementary Figure 2.1. For fluence dependence measurements, we add an additional 1.93 THz BPF before P3 to suppress the fundamental harmonic (FH). For temperature dependence of third harmonic (TH) in BSCCO(OD65), we also add an additional 1.9 THz BPF before P3.

For electro-optical sampling we used a 2 mm ZnTe crystal and 100 fs gate pulse with 800 nm central wavelength. Accelerator-based THz pump and the laser gating pulse have a timing jitter characterized by a standard deviation of ~ 20 fs. Synchronization was achieved through pulse-resolved detection (*1*).

To estimate the efficiency of the third harmonic generation (THG), the 2.1 THz bandpass filter's transmission curve should be taken into account. The measured transmission of 2.1 THz BPF is shown in Supplementary Figure 2.2:

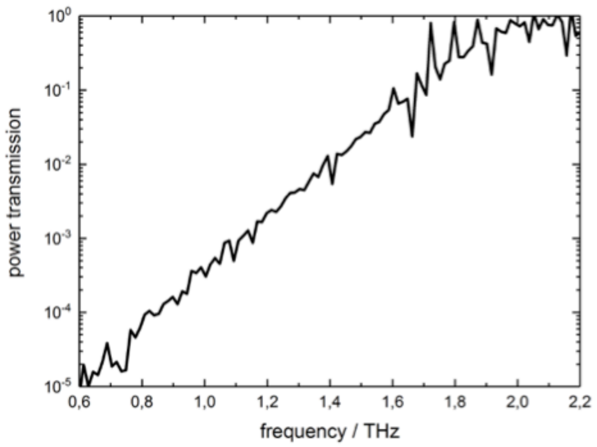

Supplementary Figure 2.2 Power transmission of 2.1 THz bandpass filter

Supplementary Note 3. Temperature dependence of FH transmission

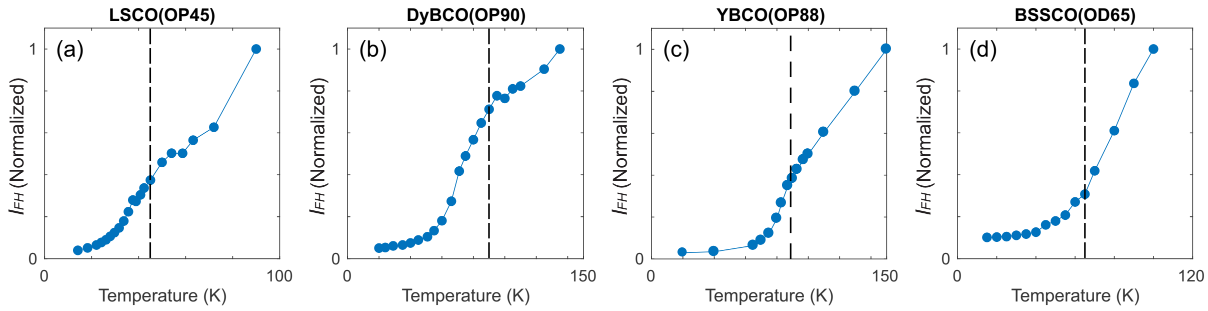

Supplementary Figure 3.1 Temperature dependence of 0.7 THz FH transmission (I_{FH}). The 0.7 THz transmission for (a) LSCO(OP45), (b) DyBCO(OP90), and (c) YBCO(OP88) are obtained from residual FH intensity in THG experiment. For (d) BSCCO(OD65), FH transmission is estimated from independent London penetration depth measurement.

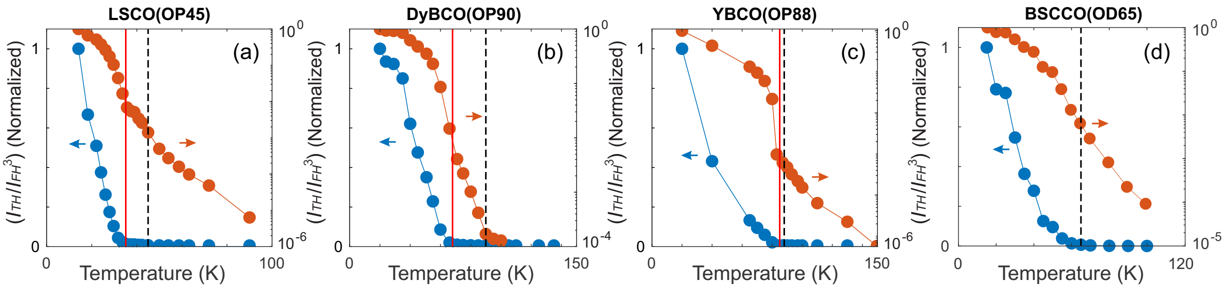

Supplementary Figure 3.2 Temperature dependence of $I_{\mathrm{TH}}/I_{\mathrm{FH}}^3$ on linear scale (blue, left axis) and log scale (orange, right axis). Black dotted line denotes T_{c}, red solid line denotes T_{π}.

During the THG experiment, we measure the residual FH intensity (I_{FH}) transmitted through the sample as a function of temperature. I_{FH} monotonically increases with temperature due to the decreasing screening effect of the superconducting condensate. For BSCCO(OD65), the transmitted FH intensity is very weak, resulting in high signal-to-noise ratio. Therefore, independent London penetration depth measurement is performed on BSCCO(OD65) to extract the transmission coefficient at 0.7 THz.

Taking the transmitted FH intensity as an estimate for the electric field inside the superconducting thin film and assuming $I_{\mathrm{TH}} \propto I_{\mathrm{FH}}^3$, we can correct for the screening effect and extract the intrinsic nonlinear response (susceptibility) of the Higgs mode. This is given by

I_{TH}/I_{FH}^3 as shown in Supplementary Figure 3.2. The absence of a resonance-like peak near T_c, in contrast to THG from *s*-wave superconductors, is consistent with a heavily damped Higgs mode in *d*-wave superconductors. Note that the dip in $I_{TH}(T)$ at T_π in LSCO(OP45) translates into a kink in $I_{TH}/I_{FH}^3(T)$. An even stronger kink is seen YBCO(OP88), while in DyBCO(OP90) it is less obvious.

Supplementary Note 4. Extraction of relative phase between TH response and FH drive

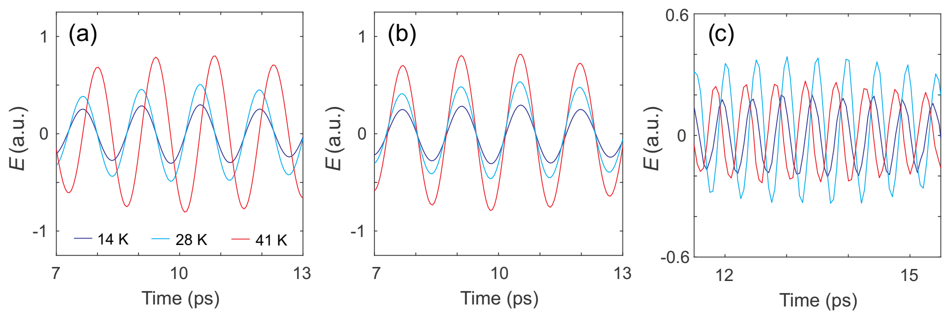

Supplementary Figure 4 Procedure for extracting the relative phase between TH response and FH drive. **a,** raw FH waveforms at a few representative temperatures below T_c in LSCO(OP45). **b,** FH waveforms aligned on top of each other after being shifted in time. **c,** TH waveforms at the same temperatures after applying the same time shift.

We first extract the raw FH and TH waveforms from the raw transmitted waveforms using 1.4 THz FFT low pass and high pass filters. For example, a few extracted FH waveforms from LSCO(OP45) are shown in Supplementary Figure 4(a). Due to the inductive response of superconductors below T_c, the FH wave experiences a phase shift across T_c on transmission through the superconducting thin film. This is also illustrated in Supplementary Figure 4(a). As a first step, we apply a time shift δt to the FH waveform at each temperature, so that their phases are all aligned with the lowest temperature waveform (Supplementary Figure 4(b)). Then, we apply the same time shift δt to the corresponding TH waveform at each temperature. The resulting TH waveforms are shown in Supplementary Figure 4(c). Any phase shift between the TH waveforms in Supplementary Figure 4(c) has to intrinsically come from the Higgs oscillation itself because the phase shift in the FH drive has already been accounted for. To extracted this relative TH phase, we fitted the waveforms in Supplementary Figure 4(c) to a Gaussian-enveloped sinusoidal function,

$$E_{\mathrm{TH}}(t) = A \exp\left(-(t-t_0)^2/c^2\right) \sin(\omega(t-t_0) - \Phi),$$

where only A, c, Φ are free fitting parameters. Φ is the relative TH phase with respect to FH drive that we discussed extensively in the main text.

Supplementary Note 5. Effects of thin film on the intensity and phase of the transmitted fields

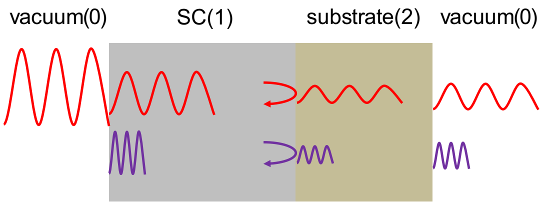

Supplementary Figure 5.1 Illustration of electromagnetic wave propagation and THG in a thin film. Transmission and reflection at each interface is described by Fresnel equations and may depend on temperature and frequency. The thickness of the superconductor (SC) film is exaggerated for illustration purpose.

In our experiment, the FH field transmits through the sample composed of the superconductor (SC) film and the substrate, while TH field is presumably generated only within the SC film. The transmission of the FH wave through the entire sample is described by the Fresnel equations

$$E_{\mathrm{measured}} = E_{\mathrm{input}} t_{01} t_1 f_1 t_{12} t_2 t_{20} \qquad (1)$$

where $t_{\mathrm{ij}} = \frac{2n_{\mathrm{i}}}{n_{\mathrm{i}}+n_{\mathrm{j}}}$ describes transmission across interface from dielectric medium i to dielectric medium j, $t_{\mathrm{i}} = \exp\left(i\omega L n_{\mathrm{i}}/c\right)$ describes transmission through dielectric medium i of length L, $f_1 = \frac{1}{1+r_{01}r_{12}\exp\left(2i\omega L_1 n_1/c\right)}$ describes Fabry-Perot effect within the superconducting film of length L_1, $r_{\mathrm{ij}} = \frac{n_{\mathrm{i}}-n_{\mathrm{j}}}{n_{\mathrm{i}}+n_{\mathrm{j}}}$ describes reflection at the interface from dielectric medium i to dielectric medium j. Here, n_{i} denotes the complex index of refraction of medium i, c denotes the speed of light in vacuum, and the subscript 0, 1, 2 refers to vacuum, SC, substrate respectively. Since n_1, the complex index of refraction of the SC film, strongly depends on temperature and frequency, transmission of the FH and TH field each develops its own temperature dependence. The measured FH and TH field outside the sample, therefore, may not be faithful representations of the FH and TH field inside the SC film. For example, the temperature dependence of I_{TH}, or $I_{\mathrm{TH}}/I_{\mathrm{FH}}^3$, using the value of I_{FH} and I_{TH} measured outside the sample may not give the real nonlinear response inside the SC film.

To account for such effect, we use the DyBCO(OP90) sample as an example and illustrate two different approaches. The first approach is based on back-calculating the electric field

immediately on the left side of the SC-substrate interface from the electric field measured outside the sample. The second approach is based on simulating the propagation of the FH wave through the sample together with the THG process inside the SC film, and then predicting how the transmitted FH and TH field outside the sample would evolve as a function of temperature. Both methods show that the thin film has negligible effect on modifying the relative strength of the FH and TH field as well as the relative phase between them.

First approach *(back-calculation)*

We are interested in knowing the exact FH and TH field inside the SC film. Since the thickness of the SC film is a very small fraction of the FH and TH wavelength and the FH and TH wave must be continuous inside the SC film, it is sufficient to know the FH and TH field, E_1^{FH} and E_1^{TH}, immediately to the left of the SC-substrate interface. The measured electric field outside the sample is given by $E_{measured} = E_1\ t_{12}\ t_2\ t_{20}$. Ignoring the temperature-independent factor ($t_2\,t_{20}$), we have $E_1^{FH} = E_{measured}^{FH}/t_{12}^{FH}$ and $E_1^{TH} = E_{measured}^{TH}/t_{12}^{TH}$, i.e. the electric field strength inside the SC film should be corrected from the electric field strength measured outside the sample by a factor $|t_{12}|$, and its phase from the measured phase by an offset $\arg(t_{12})$. Using the complex index of refraction of the DyBCO film and the LSAT substrate, we calculate t_{12}^{FH} and t_{12}^{TH}. As shown in Supplementary Figure 5.2, the amplitude correction factor $|t_{12}|$ and the phase correction offset $\arg(t_{12})$ are both small and varies little with temperature.

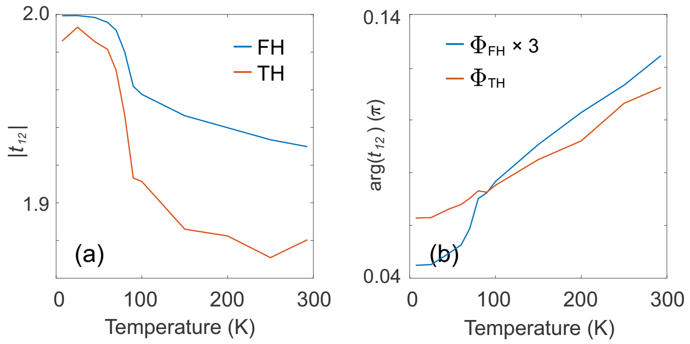

Supplementary Figure 5.2 Amplitude and phase correction for the electric field measured outside the sample due to transmission across the SC-substrate interface.

Second approach *(forward simulation)*

In this approach, we simulate the transmission of the FH wave through the entire sample and the THG process within the SC film. Below T_c, the vacuum-SC interface and the SC-

substrate interface forms two highly reflective surfaces for terahertz frequencies. Therefore, the Fabry-Perot effect needs to be considered as well. This is illustrated in Supplementary Figure 5.3. The resulting FH field inside the SC film is the superposition of all the reflected FH waves. We then assume E_1^{TH} is generated from this total FH field using the simple relation $E_1^{TH} = (E_1^{FH})^3$. The generated E_1^{TH} also undergoes multiple reflections at the two interfaces. Therefore the total TH field inside the SC film is also the superposition of all the reflections. We then calculate the field transmitted outside the sample using $E_{measured} = E_1 t_{12}$, where E_1 is the electric field immediately to the left of the SC-substrate interface. The resulting time and temperature dependence of $E_{measured}^{FH}$ and $E_{measured}^{TH}$ is shown in Supplementary Figure 5.4. It can be seen that the linear effects arising from interfaces, the SC film, and Fabry-Perot effect cause the FH and TH waves to shift a similar amount in time. This is more clearly shown in Supplementary Figure 5.5(b). The Fabry-Perot effect also leads to small correction of the electric field strength inside the SC film. Ideally, since we assumed $E_1^{TH} = (E_1^{FH})^3$ for the THG process, we should have $|E_1^{TH}/(E_1^{FH})^3| = 1$. However, $|E_1^{TH}/(E_1^{FH})^3|$ deviates from 1 due to the Fabry-Perot effect for FH and TH waves respectively. This leads to a correction factor for the FH and TH field strength inside the SC film, which, however, also turns out to be very small: Supplementary Figure 5.5(a).

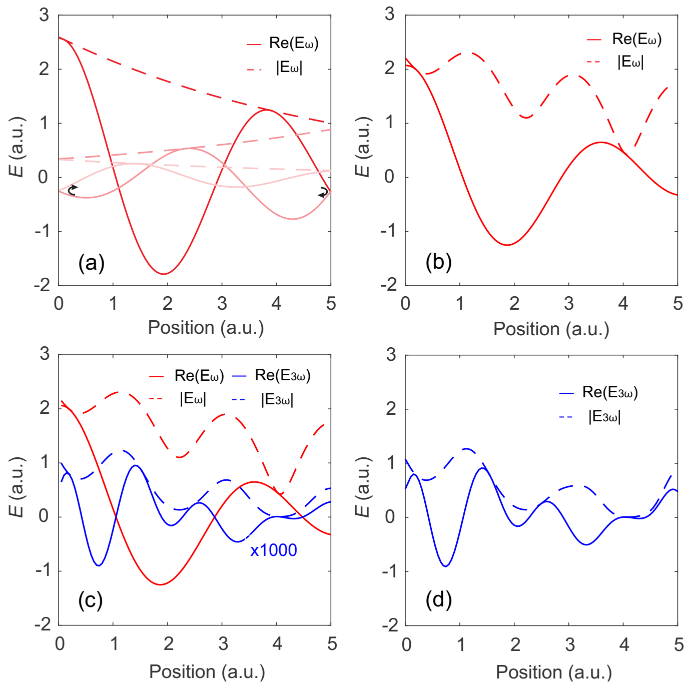

<u>Supplementary Figure 5.3</u> Illustration of FH transmission through the SC film and THG inside the SC film**. a,** multiple reflections of the FH wave inside the SC film. **b,** the resulting FH field which is the superposition of all reflected FH waves. **c,** THG from FH assuming $E_1^{TH} = (E_1^{FH})^3$. **d,** the resulting TH field which is the superposition of all reflected TH waves. For the purpose of illustration, the thickness of the SC film is exaggerated so as to accommodate 1.25 cycles of the FH wave. The actual SC film has a thickness on the order of 10^{-3} of the wavelength of the FH wave. The index of refraction is also purposely selected to illustrate absorption effect.

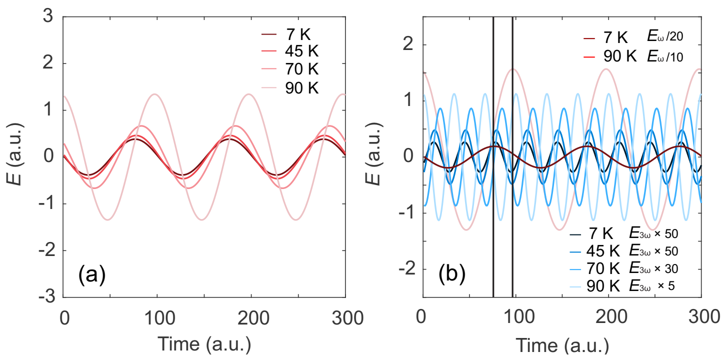

Supplementary Figure 5.4 Transmitted FH and TH fields outside the sample as a function of time. **a,** the transmitted FH field, $E_{\mathrm{measured}}^{\mathrm{FH}}$, for several different temperatures. **b,** the transmitted TH field, $E_{\mathrm{measured}}^{\mathrm{TH}}$, for the same temperatures. The transmitted FH fields at 7 K and 90 K are superposed for comparison. Both FH and TH fields undergo a similar shift in time (designated by the two solid black lines) between 7 K and 90 K.

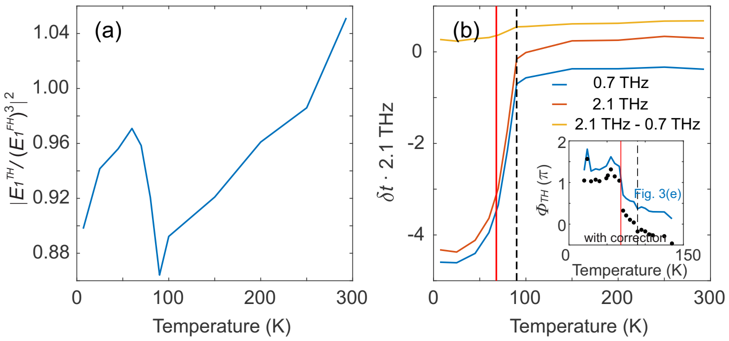

Supplementary Figure 5.5 **a,** Correction factor for $I_{\mathrm{TH}}/I_{\mathrm{FH}}^3$ due to the Fabry-Perot effect. **b,** time-shift predicted for the transmitted FH and TH waves due to temperature dependent variation of linear optical properties. It can be seen that FH and TH field undergoes a similar shift in time. Therefore, the correction for the relative TH phase coming from thin film effects (yellow curve) is quite small. In the inset, we apply this correction to the relative TH phase in DyBCO(OP90). The corrected relative TH phase (black dots) retains the sharp π jump near T_π (red solid line) similar to the original relative TH phase from the main text (blue line). Black dotted line denotes T_c.

Supplementary Note 6. Fluence dependence of THG

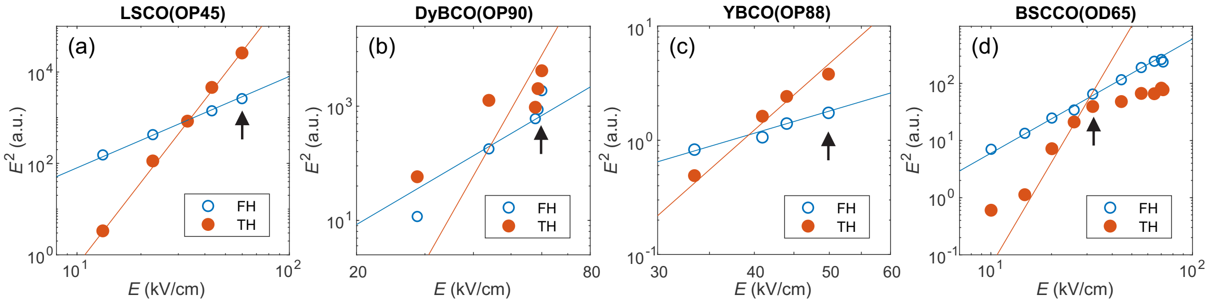

Supplementary Figure 6 Fluence dependence of THG. The transmitted TH and FH intensity is measured as a function of the incoming FH field for (a) LSCO(OP45) at 27 K, (b) DyBCO(OP90) at 70 K, (c) YBCO(OP88) at 52 K, and (d) BSCCO(OD65) at 20 K. Solid lines are guides to the eye with a slope of 2 and 6. Arrows indicate the FH field with which the data in the main text are taken.

To make sure that the THG experiment stays within perturbative regime of the Higgs mode, we performed fluence dependence measurements. As shown in Supplementary Figure 6, LSCO(OP45) exhibits excellent agreement with the $I_{TH} \propto I_{FH}^3$ dependency. YBCO(OP88) has similar $I_{TH} \propto I_{FH}^3$ dependency. BSCCO(OD65) exhibits both a $I_{TH} \propto I_{FH}^3$ regime and non-$I_{TH} \propto I_{FH}^3$ regimes. In DyBCO(OP90), deviation from the $I_{TH} \propto I_{FH}^3$ dependency is the most pronounced. Based on the results of these measurements, we pick for our experiment the highest FH field within the perturbative regime to maximize signal-to-noise ratio.

Supplementary Note 7. THG in BSCCO(OD65)

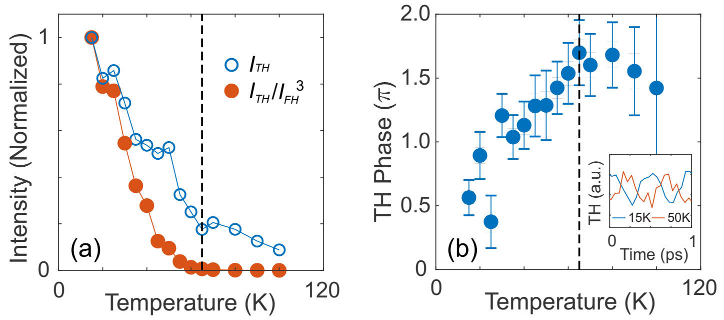

Supplementary Figure 7 Temperature dependence of TH intensity and relative phase in BSCCO(OD65).

THG from BSCCO(OD65) is very weak, which can be inferred from the noisy TH waveforms extracted in Supplementary Figure 7(b). The temperature dependence of TH intensity shows a monotonically increasing trend towards low temperature. In addition, the relative TH phase shows a gradual π shift in a direction opposite to the π phase shift in optimally doped samples. These dramatically different results compared to the optimally doped samples might be due to overdoping, but it could also be that BSCCO(OD65) exhibits a more gradual superconducting transition due to sample inhomogeneity. The inhomogeneity likely stems from an oxygen gradient that has formed in the film during the time between the sample growth and the THG measurements. For these reasons, we refrain from making interpretations about the BSCCO(OD65) THG results.

Supplementary Note 8. Driven coupled harmonic oscillators model

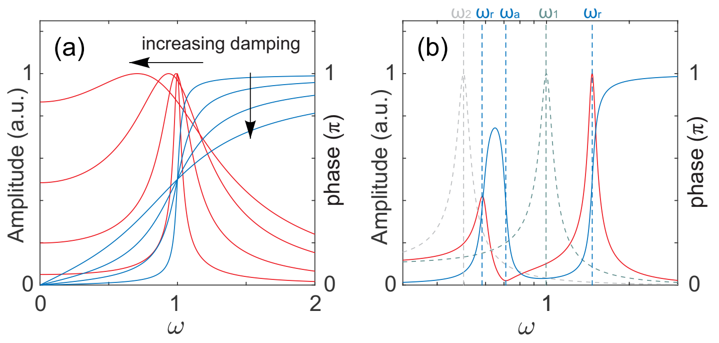

Supplementary Figure 8.1 **a,** the response of an isolated driven harmonic oscillator as a function of driving frequency. As damping increases, the peak in amplitude (red lines) moves below the resonance frequency ($\omega_1 = 1$) while the center of the phase shift (blue lines) still intersects at the resonance frequency. **b,** dotted lines show the amplitude response of two isolated underdamped driven harmonic oscillators with resonance frequency ω_1 and ω_2. Solid line show the amplitude (red) and phase (blue) response when the two oscillators are coupled. ω_r denotes the resonance frequency of the coupled system, ω_a denotes the anti-resonance frequency of the coupled system. The parameters used for generating **b** are $\omega_1 = 1$, $\omega_2 = 0.5$, $b_1 = 0.1$, $b_2 = 0.05$, $\omega_{c1} = 1$, $\omega_{c2} = 0.5$.

We consider a model of two driven damped harmonic oscillators, each modeled by a spring constant k_i, a mass m_i, a damping coefficient b_i, and a spring constant K that couples the two oscillators. Furthermore, we assume $\omega_1 = (k_1/m_1)^{1/2} = 2\Delta$, and $\omega_2 = (k_2/m_2)^{1/2} = \delta \times 2\Delta$, where $0 < \delta < 1$. Oscillator 1 represents the 2Δ Higgs mode, while oscillator 2 represents the other collective mode. Since light couples to the Higgs mode quadratically, the frequency of the periodic driving force, as felt by the Higgs mode, is 2ω. For simplicity, we drop off the factor of 2 from the driving force frequency (2ω) and the energies of the two oscillators (2Δ and $\delta \times 2\Delta$) in the following discussion. This way, the resonance condition for the Higgs mode at $2\omega = 2\Delta(T)$ is still preserved and given by $\omega = \Delta(T)$.

In the case that the two oscillators are decoupled, $K = 0$. The linear response of each oscillator is given by

$$x_i = \frac{\omega_i^2 X_i}{\omega_i^2 - \omega^2 + i b_i \omega}$$

where X_i is the amplitude of the drive, and ω is the driving frequency. A noticeable effect of damping is that it not only broadens the amplitude response, but also shifts the amplitude maximum to lower frequency: Supplementary Figure 8.1(a). However, the center of the phase shift always intersect the resonance frequency regardless of damping. Therefore, the phase response of the oscillator provides more accurate information regarding resonance, even in the event that the amplitude response becomes entirely indistinguishable, i.e. overdamped.

In the case that the two oscillators are coupled, we have additional coupling parameters defined as $\omega_{c1} = (K/m_1)^{1/2}$ and $\omega_{c2} = (K/m_2)^{1/2}$. The equation of motion for the coupled system, assuming only the Higgs oscillator is directly driven by the periodic drive, is given by

$$\begin{pmatrix} {\omega_1}^2 + {\omega_{c1}}^2 - \omega^2 + ib_1\omega & -{\omega_{c1}}^2 \\ -{\omega_{c2}}^2 & {\omega_2}^2 + {\omega_{c2}}^2 - \omega^2 + ib_2\omega \end{pmatrix} \times \begin{pmatrix} x_1 \\ x_2 \end{pmatrix} = \begin{pmatrix} {\omega_1}^2 X \\ 0 \end{pmatrix},$$

which gives the solution

$$x_1 = \frac{({\omega_2}^2 + {\omega_{c2}}^2 - \omega^2 + ib_2\omega)\cdot{\omega_1}^2 X}{\det} \quad \text{and}$$

$$x_2 = \frac{{\omega_{c2}}^2{\omega_1}^2 X}{\det},$$ where det is the determinant of the 2 × 2 matrix above.

While x_1 exhibits two resonances arising from the poles in the denominator, it also exhibits an anti-resonance arising from zero in the numerator. An example of the response of the Higgs oscillator in the coupled scenario is shown in Supplementary Figure 8.1(b). Figure 4(a)(b) in the main text is plotted with are $\omega = 0.575$, $\omega_1 = 1 \times \Delta(T)$, $\omega_2 = 0.5 \times \Delta(T)$, $b_1 = 1$, $b_2 = 0.01$, $\omega_{c1} = 1.35$, $\omega_{c2} = 0.5$, where $\Delta(T) = \sqrt{n_s(T)}$. n_s is the superfluid density in LSCO(OP45) determined from independent London penetration depth measurement. The temperature dependence of n_s for LSCO(OP45) is shown in Supplementary Figure 8.2.

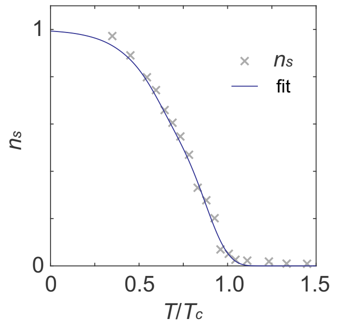

Supplementary Figure 8.2 Superfluid density in LSCO(OP45). Experimental data (crosses) are obtained from London penetration depth measurement in the terahertz and microwave frequency range. Solid line is a polynomial fit to the experimental data.

Supplementary Note 9. Extended Anderson pseudospin model

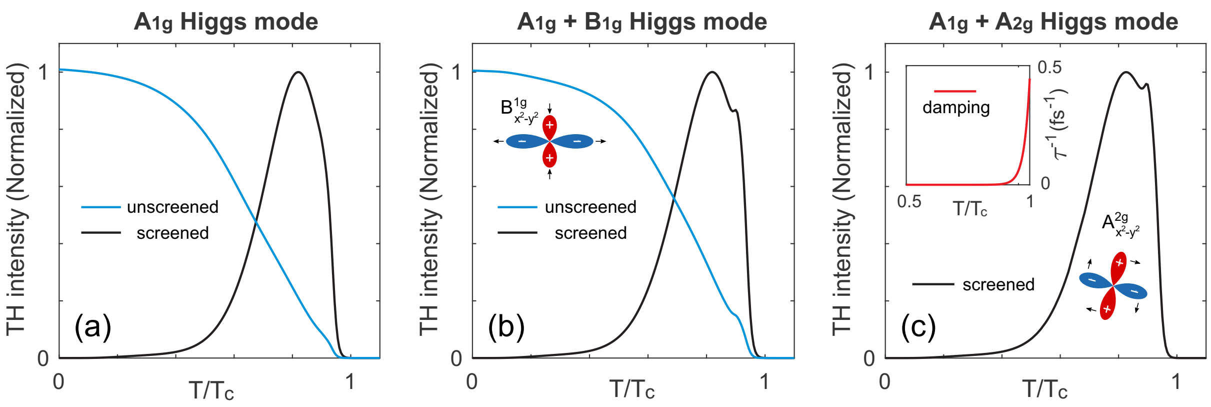

Supplementary Figure 9 Extended Anderson pseudospin model incorporating an (a) A_{1g}, (b) A_{1g} + B_{1g}, and (c) A_{1g} + A_{2g} drive of the complex order parameter Δ in the pseudomagnetic field. The inset in (c) shows the temperature-dependent damping function assumed for the model. The drawings in (b)(c) illustrate the B_{1g} and A_{2g} Higgs modes of the *d*-wave order parameter.

One potential candidate for the coupled collective is the non-A_{1g} Higgs mode of the *d*-wave superconducting order parameter. The symmetry of these modes is described as A_{2g}, B_{1g}, B_{2g}, and their motion in momentum space is illustrated in Supplementary Figure 9. More details of these symmetrical modes can be found in (*2, 3*). According to theoretical predictions, the resonance energy of the A_{2g} and B_{1g} Higgs mode is less than the A_{1g} Higgs mode. They might be potential candidate for the new collective mode. THG from the driven Higgs oscillation can be modeled by the Anderson pseudospin model. However, a coupling between the A_{1g} and non-A_{1g} Higgs mode within the Anderson pseudospin model is not straightforward. Therefore, we illustrate below the THG response of an independently driven oscillation of the A_{2g}/B_{1g} Higgs mode.

To model the TH response of a periodically driven A_{2g}/B_{1g} Higgs mode, we use an extended Anderson pseudospin formalism with a standard BCS Hamiltonian (*4-6*). The Hamiltonian reads $H = \sum_{\mathbf{k}} b_{\mathbf{k}}\,\sigma_{\mathbf{k}}$, where $\sigma_{\mathbf{k}}$ is Anderson's pseudospin describing the occupation of quasiparticles and Cooper pairs. The pseudomagnetic field reads

$$b_{\mathbf{k}} = \begin{pmatrix} -2\Delta'(t) f_{\mathbf{k}} \\ 2\Delta''(t) f_{\mathbf{k}} \\ 2\epsilon_{\mathbf{k}} \end{pmatrix},$$

with the dispersion $\varepsilon_{\mathbf{k}}$, the energy gap $\Delta(t) = \Delta'(t) + i\Delta''(t) = \frac{W}{N}\sum_{\mathbf{k}} f_{\mathbf{k}} \left(\langle\sigma_{\mathbf{k}}^{x}\rangle(t) - i\langle\sigma_{\mathbf{k}}^{y}\rangle(t)\right)$, and the gap symmetry function $f_{\mathbf{k}}$. The time evolution is described by Bloch equation $\dot{\sigma}(t) = b_{\mathbf{k}} \times \sigma_{\mathbf{k}}(t)$.

The coupling to the electromagnetic field $\mathbf{A}(t) = \mathbf{A_0} \sin(\omega t)$ is considered by minimal substitution $\epsilon_{\mathbf{k}} \rightarrow \epsilon_{\mathbf{k}-e\mathbf{A}(t)}$. To model a driving of the A_{2g} (B_{1g}) Higgs mode, we use a phenomenological approach, where the gap symmetry is modulated periodically in a different symmetry channel however with the same time-dependence as the usual driving with light. Such an asymmetric driving may result experimentally from a small in-plane component of the driving light wave vector or higher order couplings. To this end, we replace the symmetry function f_{k} in the pseudomagnetic field b_{k} by an effective time-dependent symmetry function $f_{\mathbf{k}}^{\mathrm{Q}}(t) = f_{\mathbf{k}} + \delta|A(t)|^2 f_{\mathbf{k}}^{\mathrm{Q}}$, where $f_{\mathbf{k}}^{\mathrm{Q}}$ has a different symmetry from $f_{\mathbf{k}}$. Finally, we include the screening by a temperature-dependent driving amplitude $A_0(T)$ (given by $I_{\mathrm{FH}}(T)^{1/2}$) and a temperature-dependent damping modeled by a relaxation time $\tau(T)$ in the Bloch equations (Supplementary Figure 8(c) inset). The assumption of a temperature-dependent damping follows from experimental observation of a diverging pair-breaking rate as T approaches T_c (*7, 8*).

Finally, the Bloch equation reads

$$\dot{\sigma}(t) = \begin{pmatrix} -2\Delta'(t) f_{\mathbf{k}}^{\mathrm{Q}}(t) \\ 2\Delta''(t) f_{\mathbf{k}}^{\mathrm{Q}}(t) \\ \epsilon_{\mathbf{k}-e\mathrm{A}(t)} + \epsilon_{\mathbf{k}+e\mathrm{A}(t)} \end{pmatrix} \times \begin{pmatrix} \sigma_{\mathbf{k}}^{x}(t) \\ \sigma_{\mathbf{k}}^{y}(t) \\ \sigma_{\mathbf{k}}^{z}(t) \end{pmatrix} - \frac{\sigma_{\mathbf{k}}^{z}(t) - \sigma_{\mathbf{k}}^{z}(0)}{\tau(T)}.$$

We numerically solve the Bloch equation self-consistently together with the gap equation. To obtain the THG intensity in the same polarization direction as the electromagnetic field, we evaluate $I^{\mathrm{TH}} \propto |j^{(3)}(3\omega)|^2$, where the nonlinear current is $j^{(3)}(t) \propto \Delta_0 A(t)\delta\Delta(t)$.

For the results shown in Supplementary Figure 9, we use $\delta = 0.04$ for the $A_{1g} + B_{1g}$ Higgs mode case and $\delta = 0.08$ for the $A_{1g} + A_{2g}$ Higgs mode case. The superfluid density of LSCO(OP45) is used for Δ(T).

Supplementary References:

1. Kovalev, S. *et al.* Probing ultra-fast processes with high dynamic range at 4th-generation light sources: Arrival time and intensity binning at unprecedented repetition rates. *Structural Dynamics* **4**, 024301 (2017).

2. Barlas, Y. & Varma, C. M. Amplitude or Higgs modes in d-wave superconductors. *Phys. Rev. B* **87**, 054503 (2013).

3. Schwarz, L. *et al.* Classification and characterization of nonequilibrium Higgs modes in unconventional superconductors. *Nat. Comm.* **11**, 287 (2020).

4. Anderson, P. W. Random-Phase Approximation in the Theory of Superconductivity. *Phys. Rev.* **112**, 1900–1916 (1958).

5. Matsunaga, R. *et al.* Light-induced collective pseudospin precession resonating with Higgs mode in a superconductor. *Science* **345**, 1145 (2014).

6. Tsuji, N. & Aoki, H. Theory of Anderson pseudospin resonance with Higgs mode in superconductors. *Phys. Rev. B* **92**, 064508 (2015).

7. Parham, S. *et al.* Ultrafast Gap Dynamics and Electronic Interactions in a Photoexcited Cuprate Superconductor. *Phys. Rev. X* **7**, 041013 (2017).

8. Reber, T. J. *et al.* Pairing, pair-breaking, and their roles in setting the T_c of cuprate high temperature superconductors. https://arxiv.org/abs/1508.06252 (2015).
